# *TPS* Genes Silencing Alters Constitutive Indirect and Direct Defense in Tomato

**DOI:** 10.3390/ijms19092748

**Published:** 2018-09-13

**Authors:** Mariangela Coppola, Pasquale Cascone, Simone Bossi, Giandomenico Corrado, Antonio Pietro Garonna, Massimo Maffei, Rosa Rao, Emilio Guerrieri

**Affiliations:** 1 Department of Agricultural Sciences, University of Naples Federico II, 80055 Portici, Italy; mariangela.coppola@unina.it (M.C.); giacorra@unina.it (G.C.); antoniopietro.garonna@unina.it (A.P.G.); rosa.rao@unina.it (R.R.); 2 Institute for Sustainable Plant Protection, National Research Council of Italy, 80055 Portici (Na), Italy; emilio.guerrieri@ipsp.cnr.it; 3 Department of Life Sciences and Systems Biology, University of Turin, 10135 Turin, Italy; simone.bossi@unito.it (S.B.); massimo.maffei@unito.it (M.M.)

**Keywords:** constitutive release of VOC, Sesquiterpene Synthase, RNAi, *Aphidius ervi*, *Spodoptera exigua*

## Abstract

Following herbivore attacks, plants modify a blend of volatiles organic compounds (VOCs) released, resulting in the attraction of their antagonists. However, volatiles released constitutively may affect herbivores and natural enemies’ fitness too. In tomato there is still a lack of information on the genetic bases responsible for the constitutive release of VOC involved in direct and indirect defenses. Here we studied the constitutive emissions related to the two most abundant sesquiterpene synthase genes expressed in tomato and their functional role in plant defense. Using an RNA interference approach, we silenced the expression of *TPS9* and *TPS12* genes and assessed the effect of this transformation on herbivores and parasitoids. We found that silenced plants displayed a different constitutive volatiles emission from controls, resulting in reduced attractiveness for the aphid parasitoid *Aphidius ervi* and in an impaired development of *Spodoptera exigua* larvae. We discussed these data considering the transcriptional regulation of key-genes involved in the pathway of VOC metabolism. We provide several lines of evidence on the metabolic flux from terpenoids to phenylpropanoids. Our results shed more light on constitutive defenses mediated by plant volatiles and on the molecular mechanisms involved in their metabolic regulation.

## 1. Introduction

During their life cycle, plants interact with the surrounding environment by producing secondary metabolites including volatile organic compounds (VOC). Volatile organic compounds cover multiple biological and ecological functions that provide reproductive advantages, for example by protecting plant from pests [1,2,3] or acting as airborne “warning” signals and infochemicals within the plant and in plant communities [4,5]. Protection may be exerted directly by affecting herbivore’s preference and performance on the host plant [6,7] or indirectly by recruiting natural enemies that attack herbivores reducing their fitness, and consequently, their negative impact on infested plants [8]. Plants are able to synthesize a wide array of volatiles which are mainly represented by terpenoids, fatty acids and amino acid derivatives, including those originating from L-phenylalanine (Phe), phenylpropanoids, and benzenoids. Terpenoids are the most represented compounds in the VOC blend emitted by plants upon insect injury and they play a crucial role in the attractiveness of the third trophic level (*i.e.*, insect parasitoids and predators) [8]. For example, tomato plants infested with the potato aphid *Macrosiphum euphorbiae* release a blend of volatiles with increased amount of terpenoids that attract the aphid parasitoid *Aphidius ervi* [9]. Similarly, tomato cultivars increase the emission of mono and sesquiterpenoids two hours after *Spodoptera litura* feeding [10], while in maize, foliar damage caused by lepidopteran larvae induces the release of a complex volatile blend enriched in terpenoids molecules involved in the attraction of the specific herbivore antagonists [11]. Moreover, in *Medicago truncatula* leaves, *Spodoptera exigua* damage increases the transcriptional levels of *terpene synthase* (*TPS*) genes [12] corroborating the general assumption that terpenoids are regulated by the modulation of *TPSs* gene transcription [13]. Since the seminal study of Price and collaborators [14] who defined the third trophic level “as a part of plant’s battery of defenses against herbivores” (termed indirect defense), many researches focused on volatiles induced upon insect injury and on their role in plant protection [15,16]. However, more recently, the suggestion that the constitutive volatile emissions from undamaged plants may be an adaptive aspect of the overall plant indirect defense has been proposed [17]. In tomato plants, considerable progress has been made in the characterization of VOC in relation to indirect defenses [18,19,20,21,22] although the complete scenario of the genes involved in the production of these volatiles and their regulation are far from being completely understood. Like other crops, in tomato the variability of both constitutive and induced volatiles emission patterns is controlled by the genetic background: different tomato cultivars show both quantitative and qualitative differences in the VOC blend before and after herbivore induction [10]. The tomato genome contains a terpene synthase gene family composed by at least 29 potentially functional members [23]. Two closely linked *TPS* genes, *TPS9* and *TPS12*, encode for two enzymes involved respectively in the formation of the sesquiterpenes germacrene C, β-caryophyllene, and α-humulene from farnesyl pyrophosphate (FPP) [23,24]. These genes are responsible for the majority of the sesquiterpenes found in the leaf trichomes that efficiently attract natural enemies in different multitrophic systems [9,25]. Therefore, it is reasonable to hypothesize an involvement of *TPS* genes in the constitutive production of volatiles responsible for the attractiveness towards the third trophic level. To address this hypothesis, we studied the impact of the downregulation of the closely related *TPS9-12* genes on the volatile blend composition and the performance of tomato transgenic plants towards direct and indirect defenses. A similar approach based on single-step metabolic engineering has been successfully used to uncover gene functions [26,27,28,29]. For example, the genetic transformation of *Arabidopsis thaliana* with *TPS* genes, resulted in the emission of novel volatiles compounds highly attractive for herbivore antagonists [26,27,28,29]. Although it is difficult to predict the detailed outcome of a specific alteration of the pathway of secondary metabolites and how these modifications will affect plant-environment interactions, we provide a more comprehensive understanding of the regulation of the volatile blend emission in tomato plants.

## 2. Results

### 2.1. Development of Transgenic Tomato Lines and Confirmation of TPS Genes Silencing

**Figure 1:**
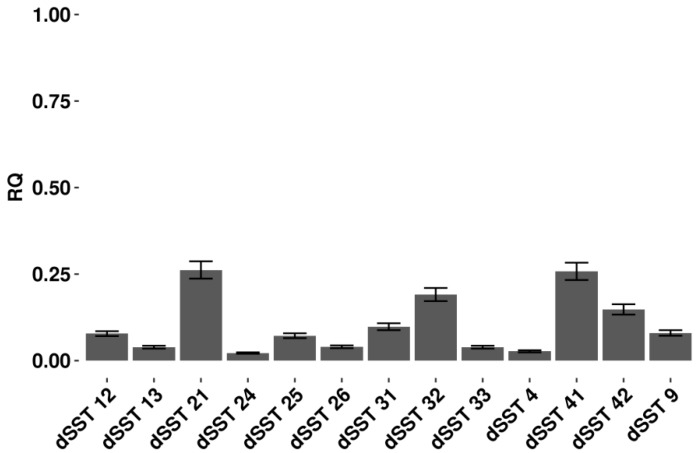
Relative quantification (RQ) of the *TPS9* and *TPS12* gene expression using real-time RT-PCR. Bars show the RQ for target genes in dSST T0 plants relative to the un-transgenic calibrator cv Red Setter (RQ = 1, not showed). On the x-axis are reported the plant lines analyzed. On the y-axis is reported the result of the relative quantification and standard error (*n* = 8). Relative quantities are graphed on a linear scale. All 2^−ΔΔCt^ values were significantly different (*p* < 0.01; Student’s *t*-test).

To study the biological functions of *TPS9* and *TPS12* genes, we suppressed their expression in tomato plants by RNA interference approach [30]. To this aim we cloned a fragment of 401 bp amplified from a region common to both cDNAs in sense and antisense orientation in to the pHannibal plasmid provided by The Commonwealth Scientific and Industrial Research Organization (CSIRO). Antisense cassette was then subcloned in a plant expression vector and used to transform tomato through *Agrobacterium tumefaciens*. Plant genetic transformation yielded 22 primary transformants, numbered as dSST plants, that did not display any phenotypic alteration. The expression of the target genes was quantified in thirteen independent transformants. Figure 1 shows that there was an efficient silencing in T0 generation of all analyzed transgenic lines since the expression of *TPS* genes varied from 0.02 up to 0.26 with respect to untransformed control plants. Two transgenic genotypes (dSST26 and dSST33) among those with the lowest relative quantification (RQ) values (Figure 1) and with the highest number of seeds available were selected for further characterizations of their T1 progenies. No phenotypic alterations were observed for T1 generation four and eight weeks after sowing (see Supplementary Figure S1).

### 2.2. TPS Genes Silencing Compromises Indirect Defence

**Figure 2:**
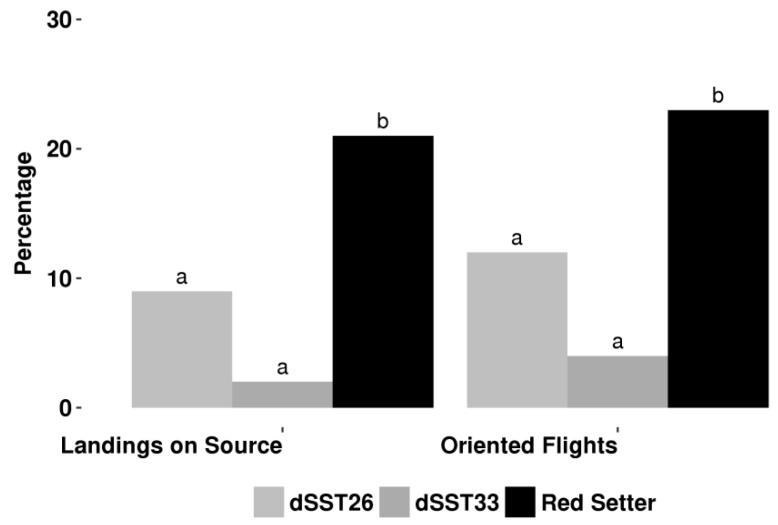
*Aphidius ervi* wind tunnel. Percentage of females showing oriented flights and landings on the target for dSST and control plants. For each test, 100 females were sampled. Different letters indicate significant differences between means (G-test, *p* < 0.05).

To test whether *TPS* genes’ silencing alter indirect defenses, we analyzed the behavioral response of the aphid parasitoid *Aphidius ervi* when exposed to dSST plants using a wind-tunnel assay and a four way-olfactomer. In a wind tunnel, dSST plants were significantly less attractive towards the parasitoid with respect to the untransformed control (cv Red Setter) in terms of both oriented flights and landings on the source in wind tunnel bioassay (Figure 2).

**Figure 3:**
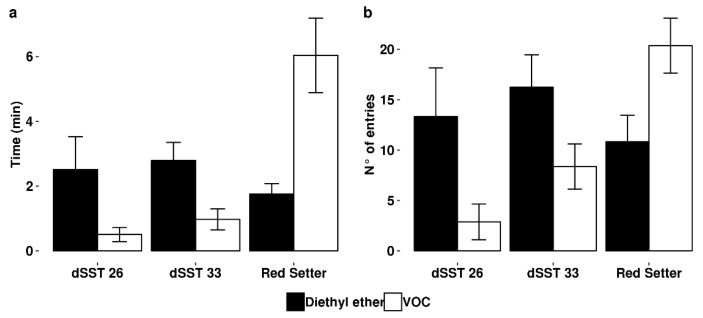
*Aphidius ervi* four-way olfactometer. Time spent and (**a**) number of entries (**b**) of adult females attracted by volatiles collected from Red Setter and dSST plants and control ethyl ether. Error bars represent ± SE (*n* = 10). All values were significantly different (*p* < 0.01; Student’s *t*-test).

Similarly, we observed that female parasitoids did not prefer the volatiles collected from dSST plants in olfactometer bioassay. Indeed, both the time spent and the number of entries in the arm containing VOC from dSST plants were strongly reduced in comparison to those recorded for the blend collected from untransformed control plants (Figure 3).

To investigate whether the observed behavioral differences relate to plant volatile blend composition, we analyzed and compared the VOCs emitted by dSST and untransformed control plants. We found that the reduced expression of *TPS9-12* genes modified the composition of the constitutive blend of volatile compounds, altering mainly those belonging to phenylpropanoid/benzenoids pathways, with direct consequences on the attraction of the parasitoid *A. ervi. TPS* gene silencing caused qualitative and quantitative changes in the volatile emission, in particular among benzenoids group (Table 1).

**Figure 4:**
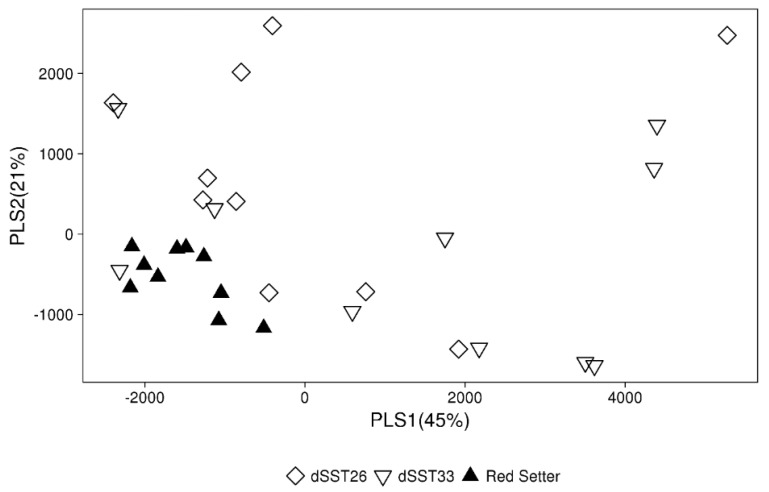
Partial least squares discriminant analysis (PLS-DA). Loading plots of the first two components based on the volatile emission of dSST and control plants. In brackets the percentage of variation explained is indicated.

Partial least squares discriminant analysis (PLS-DA) analysis on VOCs corroborated the overall differences between dSST and control plant (Figure 4).

We hypothesized that the observed differences were due to a transcriptional regulation between the terpenoid and phenylpropanoid metabolic branches to be responsible for controlling their overall production. Thus, an alteration in one pathway may have an effect on the production of metabolites from the other pathway. To verify this hypothesis, we carried out transcriptional analysis using microarray technology.

### 2.3. Transcriptomic Analyses

The transcriptomic changes determined by *TPS* gene silencing in tomato plants were monitored using the Tomato Gene Expression array (Agilent™ 4 × 44k). RNA from the dSST26 and dSST33 plants were compared with RNA of “Red Setter” plants. Most of the differentially expressed sequences had very similar expression patterns and therefore, with the purpose to avoid any position effects, the two genotypes were analyzed jointly. Differentially expressed RNAs were identified according to the Benjamini and Hochberg False Discovery Rate (*p*-value < 0.05) to minimize selection of false positives. Only sequences with rates lower than 0.05 and fold change equal to or greater than twice the fold change of control were identified as differentially expressed and considered for further investigations. This analysis identified 191 unique transcripts differentially expressed in dSST genotypes; among them 35.2% were upregulated, while 64.8% were down-regulated. Fourteen sequences were selected for validation of the expression level by real time RT-PCR. The results, shown in Supplementary Figure S2, were consistent and highly correlated with microarray data. Differentially expressed sequences were functionally annotated using Blast2GO software (http://www.blast2go.org), (Table S1), and KEGG (Kyoto Encyclopedia of Genes and Genomes) Automatic Annotation Server (http://www.genome.jp/kegg/kaas/) reported in brackets within the text.

**Figure 5:**
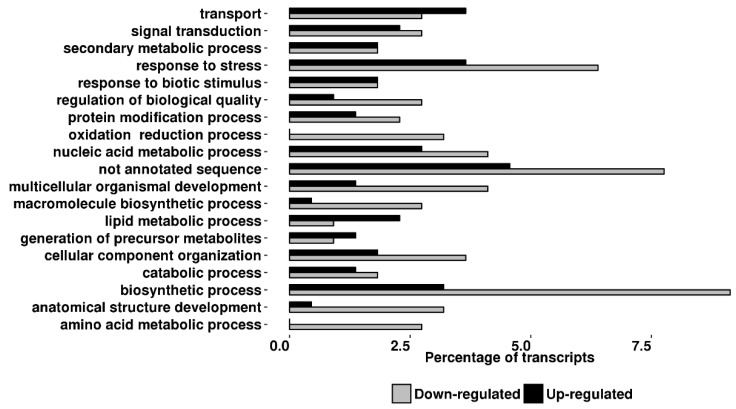
Gene ontology distribution. Terms for differentially expressed genes in dSST plants are based on the ontological domain biological process. The distribution of categories is given as a percentage of the total 234 differentially expressed transcripts (*p* ≤ 0.01).

**Figure 6:**
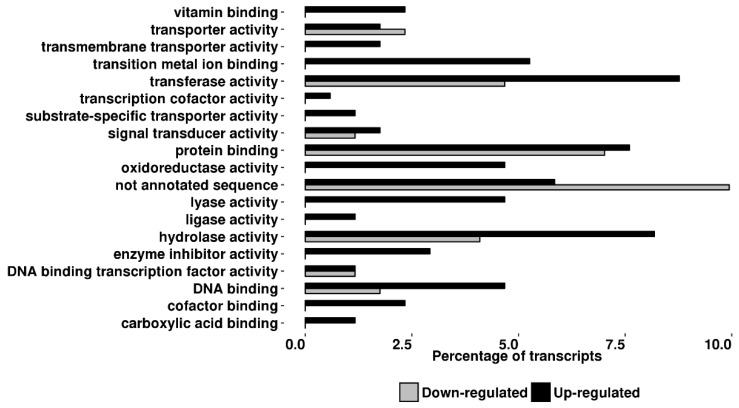
Gene ontology distribution. Terms for differentially expressed transcripts in dSST plants are based on the ontological domain molecular functions. The distribution of categories is given as a percentage of the total 234 differentially expressed transcripts (*p* ≤ 0.01).

The distribution of both up- and downregulated transcripts according to gene ontology (GO) categories, biological process (BP), and molecular function (MF) are shown in Figure 5 and Figure 6.

Genes with BP related to biosynthetic processes (12.3%) and response to stress (10%) represent major categories whose components are deregulated following RNAi of *TPS* genes while the most relevant differences in the MF categories related to several different enzymatic activities. Overall, the gene ontology analysis showed the alteration of several biological and molecular functions suggesting an alteration in the cross-talk among different biosynthetic pathways. Among the upregulated transcripts we found the genes coding for six transcripts coding for: peroxidase (*PXD*), flavonoid 3′,5′-hydroxylase (*F35H*), putative anthocyanin permease (GenBank: AY348872), disease resistance response protein 206-like (GenBank: BF097728), pathogenesis-related protein 5-like (GeneBank: ES895666), udp-glucose: flavonoid glucoside–glucosyltransferase (GenBank: AK329954), all active in the flavonoids/anthocyanins branch pathway. Peroxidase (*PXD*) belongs to the class III peroxidases (EC 1.11.1.7) involved in cell wall metabolism, lignification and defiance against pathogens; *F35H* is an hydroxylase (EC 1.14.13.88), a key enzyme involved in the first step of the branch leading to anthocyanins synthesis [31]; AY348872 is an anthocyanin permease acting as vacuolar transporter in tomato leaves [32]; BF097728 contains a conserved dirigent-like protein domain involved in the biosynthesis of lignans, flavonolignans, and alkaloids and thus playing a central role in plant flavonoids metabolism. The EST sequence ES895666 encodes a conserved domain of thaumatin-like proteins whose activities are related to plant resistance against pathogens [33]; AK329954 transcript is a flavonoid glucoside glucosyltransferase, an enzyme involved in anthocyanins production [34]. Among the downregulated transcripts we found those coding for enzymes active in the Phenilpropanoids/Benzenoids pathway such as Phenylalanine Ammonia Lyase (*PAL*), a key enzyme of the initial step of benzenoids production and a transcript coding for a benzoic acid/salicylic acid methyltransferase (Genbank: ES894405) similar to *BSMT* genes found in petunia, also involved in benzenoids pathway [35].

**Figure 7:**
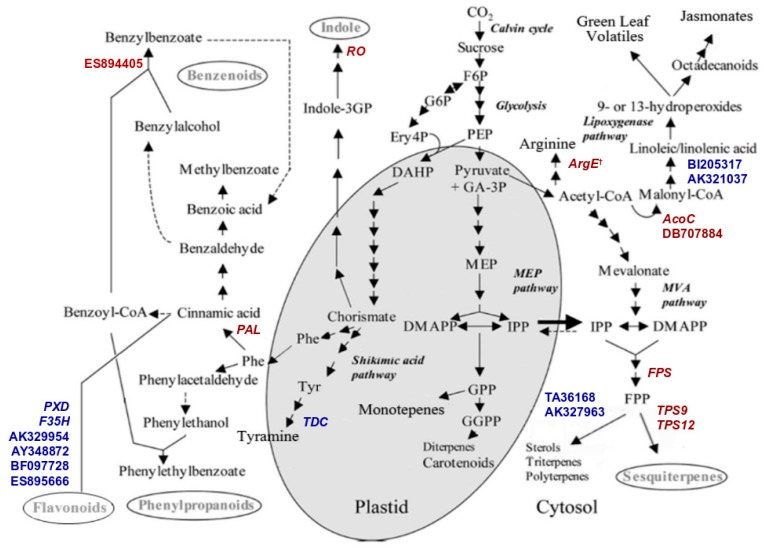
Metabolic pathways leading to the biosynthesis of VOC in plants (modified from Dudareva et al. 2006). Upregulated transcripts are in blue, downregulated in red. Abbreviations: Acetyl-CoA, acetyl coenzyme-A; DAHP, 3-deoxy-d-arabino-heptulosonate 7-phosphate; DMAPP, dimethylallyl diphosphate; Ery4P, erythrose 4-phosphate; F6P, fructose 6-phosphate; FPP, farnesyl diphosphate; GA-3P, glyceraldehyde-3-phosphate; G6P, glucose 6-phosphate; GPP, geranyl diphosphate; GGPP, geranylgeranyl diphosphate; Indole-3GP, indole 3-glycerol phosphate; IPP, isopentenyl diphosphate; MEP, 2-C-methyl-D-erythritol 4-phosphate; MVA, mevalonate; PEP, phosphoenolpyruvate. Enzyme abbreviations: *Phe*, phenylalanine; *Tyr*, Tyramine; *PAL*, phenylalanine ammonia lyase; *PXD*, peroxidase; *FPS*, farnesyl diphosphate synthase; *F35H*, flavonoid 3-hydroxylase; *RO*, Reticulin Oxidase, *TDC*, Tryptophan Decarboxylase; *AcoC*, acetyl-carboxylase; *ArgE*, Acetylornithine deacetylase, *TPS*9-12: Terpene Synthase 9-12.

In addition, the genome wide expression analysis highlighted four differentially expressed transcripts that encode enzymes active in the lipid metabolism and strictly related to the green leaf volatiles/jasmonate pathway (Figure 7). Two of them were down-regulated: the transcripts encoding acetyl-CoA carboxylase (*AcoC*, EC:6.4.1.2) and a β-Ketoacyl-synthase (Genbank: DB707884, EC:2.3.1.-); they synthesize Malonyl-CoA from acetyl-CoA, playing an important role in the elongation of fatty acid and biosynthesis of jasmonates. On the contrary, two transcripts annotated as encoding for Δ-12 fatty acid desaturase (Genbank: BI205317, AK321037) enzymes involved in the jasmonic acid pathway, resulted upregulated.

Other differentially expressed transcripts encode enzymes involved in different branches of VOC pathways, and reticulin oxidase (*RO*), Acetylornithine deacetylase (*ArgE*, EC:3.5.1.16) and Tryptophan decarboxylase (*TDC*, EC:4.1.1.25). Reticulin oxidase is involved in Alkaloids/Indole metabolism, *ArgE* is involved in arginine biosynthesis starting form glutamate and Acetyl CoA [36], and *TDC* is involved in the production of Tyramine. Beside *TPS9* and *TPS12*, also Farnesyl PyrPophosphate Synthase (*FPS1*) resulted down-regulated. Conversely, transcripts encoding enzymes involved in sterols and polyterpene pathways, that use the same common substrate of sesquiterpene synthase (Farnesyl Pyrophosphate), annotated as 24-Sterol c-Methyltransferase (Genbank: TA36168, EC:2.1.1.143) and Dolichol Kinase (Genbank: AK327963, EC:2.7.1.108) were up-regulated. Finally, dSST plants showed the downregulation of genes encoding for Aminocyclopropane Carboxylate Synthases (*ACS* and *ACS3*) enzymes, involved in the production of ethylene and associated with benzenoids and phenylpropanoids regulation [37,38].

Overall, these data suggest that, following *TPS* genes silencing, dSST plants underwent a metabolic reconfiguration involves transcriptional regulation and a tight linkage between terpenoids and shikimate/benzenoids pathways, similarly to what reported for other plant families [39]. Based on the functional annotations obtained by blast2go and KAAS (KEGG Automatic Annotation Server, http://www.genome.jp/kegg/kaas/) analysis, we located the enzymes which are differentially expressed in dSST plants in comparison with control plants on the model previously published by Dudareva and co-workers (2006) [40] to illustrate the interpretation of our results (Figure 7). The reduction in keys benzenoids emission (e.g., benzaldehyde, generally involved in indirect defenses), upon *TPS9* and *TPS12* silencing could be explained through the regulation of key genes coding for enzymes active on the same substrate, so that the shift to a different metabolic direction might be influenced by the abundance of the common substrate. Starting from terpenoids branch, silencing of *TPS* genes coding for sesquiterpene synthase causes an increment of Farnesyl Diphosphate (FPP), that triggers the downregulation of FPS gene and the overexpression of genes involved in the sterols pathway that use the same substrate (FPP). Following upstream the Mevalonate pathway, dSST plants reconfigure also, through transcriptional regulation, the linoleic acid metabolism by upregulating genes involved in its biosynthesis (*i.e.*, omega-6 fatty acid desaturase) and reducing the expression of enzymes (*i.e.*, *ArgE* and *AcoC*) which compete for a relative common substrate (Acetyl-CoA). On the other side, a similar gene transcriptional regulation on the Phenylpropanoid pathway lead us to postulate a metabolic flux for Flavonoids metabolism at the expense of Benzenoids. In fact, we found a downregulation of *PAL* and *BSMT* like genes that are involved in the early and late step of the benzenoids production respectively. Our results and previous observations that the flux of carbon source should be greater through the core phenylpropanoid pathway than in the pathway that produces volatile compounds derived from *t*-cinnamic acid [37], It appears that, dSST plants redirect carbon source from terpenoids/jasmonates to other pathways such as phenilpropanoids/benzenoids through a transcriptome reconfiguration.

### 2.4. TPS Genes Silencing Influences Direct Defence

**Figure 8:**
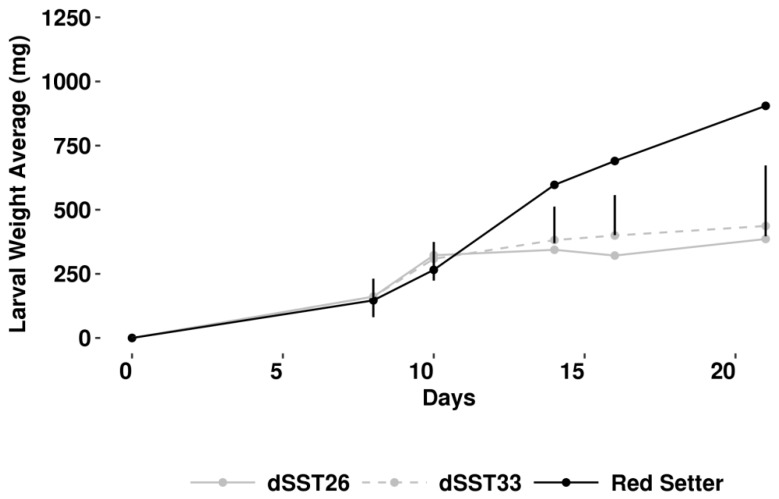
*Spodoptera exigua* bioassay. Average weight of larvae feeding upon detached leaves of dSST plants and control ones; the vertical bars represents the standard errors: significant level of probability by Fisher's least significant difference test.

Flavonoids and anthocyanins genes play a pivotal role in plant direct defense, especially against chewing herbivores [41,42,43]. The genome wide expression analysis of highlighted several genes upregulated, involves in their synthesis, in dSST plants. Thus, we hypothesized that TPS gene silencing can alter also direct defense. To verify this, we assessed the performance of *Spodoptera exigua* on dSST plants, using a no-choice feeding bioassay. We observed a significant decrease of larval weight after 21 days of feeding on detached dSST leaves in respect to larvae fed on untransformed control (Figure 8). The reduced larval weight appears to be the consequence of the transcriptome reprogramming occurred in dSST plants.

## 3. Discussion

Volatiles released by plants after pest injury have been often demonstrated to play a crucial role in triggering the so called “indirect induced defense” resulting in the increased attraction of herbivore enemies [11,26,28]. Much less is known about the role of VOCs emitted constitutively by plant, because rarely these have been studied separately from the herbivore-induced ones. Indirect defenses increase has been documented for uninfested plants attracting aphid parasitoids (reviewed by Hatano [44]) and two lepidopteran parasitoids [45,46]. In tomato there is still a lack of information on the genetic bases responsible for the constitutive release of VOC, while it has been observed that different cultivar can exhibit a different level of the indirect defenses possibly due to different VOC blends released [10,47,48]. In fact, it was demonstrated that insect parasitoids could learn about the amount and quality of the volatiles released constitutively by uninfested plants during an oviposition experience, developing a successful strategy to find their host [17]. In our test, plants silenced for the expression of two closely related *TPS* genes resulted less attractive towards *A. ervi* females than control plants. The analyses of the VOCs released by silenced plants suggested that what observed is due to a reduced amount of different volatiles emitted (Table 1). Yet, Wei and collaborators provided an evidence of the trade-off between direct and indirect defenses demonstrating a relationship between the increased resistance against *Liriomyza huidobrensis* and the reduced attractiveness towards parasitoid females [49]. Our results are in line with these findings, because in dSST plants we recorded a significant lower larval weight of *S. exigua* (2-fold decrease) (enhancement of direct defense) associated to a lower attractiveness towards the parasitoid *A. ervi* (reduction of indirect defense). Transgenic plants analyzed here showed a reduced emission of both benzaldehyde, 2,4 dimethyl benzaldehyde, which are strong attractants for *A. ervi* [50,51]. This VOC reduction is explained by the down-regulation of *PAL* and ES894405 transcripts: the former is the he first enzyme involved in the phenilpropanoids pathway, while the latter is annotated as benzoic acid/salicylic acid methyltransferase and its translated protein share 63% identity 76% similarity with *BSMT* genes in petunia and 43% identity 59% similarity with *BAMT* in snapdragon. Both transcripts were down-regulated in dSST plants analyzed in our work. Accordingly, it was previously demonstrated that their orthologs are responsible for the production and regulation of benzenoids emission [52,53,54]. Therefore, it is reasonable to associate the down-regulation of *PAL* transcripts with a reduction of several benzenoids such as benzaldehyde and 2,4 dimethyl benzaldehyde. In fact, it was reported that, in plant carbon flux toward shikimate pathway is also regulated at the transcriptional levels [55]. We have a direct evidence of transcriptional regulation of enzymes involved in VOC pathway (Figure 7) beside gene onthology categorization showing that the biosynthetic process largely involved. However, we cannot exclude that not annotated transcripts, representing the second most abundant category in Figure 5 and Figure 6, could play a role in metabolism of VOCs.

In basil, it was demonstrated that terpenoids and shikimate pathways can compete for carbon source by analyzing different lines able to regulate the carbon flux through the differential gene expression at major metabolic branch points [39]. Here we have indirect evidence of a carbon flux from terpenoids to phenylpropanoids, as demonstrated by finding several genes upregulated in flavonoids branch, and by the lower weight of larvae feeding on tomato transgenic leafs. This could be explained by an accumulation of flavonoids/anthocyanins compounds at the expense of terpenoids in dSST plants. Even if we didn’t characterize the metabolic compounds produced by dSST plants, especially flavonoids content, we can exclude that the effect against *Spodoptera* larvae was due to the production of anti-herbivore compounds like proteinase inhibitor and/or polyphenol oxidase because the array analysis didn’t display any significant differences for the relative transcripts (Table S1). In fact, transcripts generally involved in herbivory defense, including (U50152.1) Leucine Aminopeptidase (Genbank: U50152.1), (BE354788.1) Kunitz-type Protease Inhibitor Precursor (Genbank: BE354788.1) and (TC204487) Proteinase Inhibitor II (Genbank: TC204487), were down-regulated in dSST plants. We thus conclude that the reduced larval weight of *S. exigua* larvae fed on transgenic leaves was related to the upregulation of genes belonging to flavonoid/anthocyanin biosynthesis. The carbon flux is redirected following an accumulation of FPP, due to both sesquiterpene silencing and down-regulation of the enzyme who utilizes Co-enzyme A to synthetize Malonil-CoA. These two braches could lead to an increase of Phosphoenolpyruvate (PEP) through Mevalonate (MVA) pathway. In tomato plants, a redirection towards phenilpropanoids synthesizing 3-deoxyDeoxy-d-arabino-heptulosonate 7-phosphate (DAHP) from PEP and erythrose-4-phosphate (E4P) pathway occurred as reported for tobacco, where silencing trans-ketolase (enzyme responsible for E4P synthesis) has a dramatic effect on phenylpropanoids metabolism [56]. Although further characterization of dSST plant needed to demonstrate that the metabolism reconfiguration is linked to a substrate availability, here we report for the first time a tightly transcriptional regulation between terpenoids and phenylpropanoids allowing tomato plant to switch between direct and indirect constitutive defense.

## 4. Material and Methods

### 4.1. Genetic Construct and Tomato Transformation

A 401 bps sense fragment of tomato cDNA was amplified with the following primers:

5′-CGCGGATCCCATGGAAGGACATAAACAAACAA-3′

5′-CCCAAGCTTTCCAAACTCACAAGCTGGAA-3′

and the following primers for the anti-sense fragment:

5′-CCGCTCGAGCATGGAAGGACATAAACAAACAA-3′

5′-CGGGGTACCTCCAAACTCACAAGCTGGAA-3′

PCRs were carried out using thermal cycler iCycler (Biorad, Milan, Italy). Reactions (total volume of 50 μL) were prepared with 5 μL of 10X *Pfx* Amplification Buffer, 1 µL of 50 mM MgSO_4_, 1.5 µL of 10 mM dNTP mixture, 2 units of Platinum^®^ *Pfx* DNA Polymerase (Invitrogen, Milan, Italy), and 0.3 mM of each primer. The thermal cycling program started with a step of 2 min at 94 °C, followed by 30 cycles of a 15 s step at 94 °C, 30 s at Ta indicated in Table 2, and 1 min at 68 °C. The amplified sequence was cloned into both forward and reverse orientations flanking the Pdk intron of the pHANNIBAL [1] vector following standard gene cloning methods [57]. After construction and verification by sequencing, the expression region was excised from recombinant plasmid with *Not*I and then subcloned blunt-end into pBIN19 [58] cuts with *Sma*I restriction enzyme, for transformation of the Agrobacterium strain LBA4404 by the freeze-thaw method. Tomato (*Solanum licopersicum* cv Red Setter cotyledons were transformed as described [59], except that the time of co-cultivation was of 10 min and no feeder layer was used. Putative transformants were selected on Kanamycin containing (50 µg/mL) substrate. The presence of the RNAi construct in the Kanamycin resistant plant lines was confirmed by PCR with primers 5′-GAAGCAAGCCTTGAATCGTC-3′ and 5′TGCTGACCCACAGATGGTTA-3′ designed to amplify the cauliflower mosaic virus 35S^2^ promoter region of the introduced DNA. Selected transformants, named and numbered as dSSTxx, were self-pollinated to produce T1 generations. Transformed T1 and relative control tomato seeds (cv. Red Setter) were surface-sterilized with sodium hypochlorite, rinsed, washed with 70% ethanol and rinsed five times with sterile distilled water. Seeds were germinated on wet sterile paper in the dark in an environmental chamber at 24 °C. Upon root emergence, plantlets were transferred to sterile soil in an environmental chamber at 26 ± 2 °C with a photoperiod of 16/8 h light/dark.

### 4.2. RNA Extraction, cDNA Synthesis and Real-Time RT-PCR Analysis

Total RNA isolation from leaves and first-strand cDNA sythesis were performed according to previously reported procedures [60]. Amplification of the cDNA coding for the Elongation Factor 1-α gene, an ubiquitously expressed gene [61], served as control for cDNA synthesis and PCR efficiency in the different samples. The absence of genomic DNA contaminant molecules were verified by PCR amplification with two primers PcEF1FwRt and PcEF1RwRt localized in two contiguous exons [62]. Real-time PCRs were carried out by using the ABI PRISM 7000 Sequence Detection System (Applied Biosystems, Monza, Italy). Reactions (total volume of 25 μL) were prepared with 12.5 μL of the 2× SYBR Green PCR Master Kit (Applied Biosystems, Monza, Italy), 0.7 pmol of a primer pair, and 0.4 μL of cDNA template. Three independent amplifications were performed from each cDNA sample, and experiments were done in triplicates. The thermal cycling program started with a step of 2 min at 50 °C and 10 min at 95 °C, followed by 40 cycles of a 15 s step at 95 °C followed by 1 min at Ta indicated in Table 2.

The amplification products were also resolved onto agarose gel to verify amplicon size. The primer pairs used and the size of the expected amplicons are illustrated in Table 2. Primers, designed with the aid of the Primer Express 2.0 software (Applied Biosystem, Monza, Italy) were chosen to amplify a fragment between 50 and 150 bp. Relative quantification of gene expression was carried out using the 2^−ΔΔCT^ method [63], where ΔCt = Ct_target gene −_ Ct_reference gene_. We used the housekeeping Elongation Factor 1-α gene [61,64] as an endogenous reference gene for the normalization of the expression levels of the target genes. The statistical significance of the results was evaluated as already reported [65].

### 4.3. Indirect Defence Bioassay

The level of indirect defenses in terms of attractiveness towards the parasitoid *Aphidius ervi* was assessed in Y-tube olfactometer and Wind Tunnel biossay. The parasitoid was permanently reared on the pea aphid *Acyrtosiphon pisum* reproduced on broad bean plants (cv Aquadulce) from material collected in the field (Battipaglia, SA, Italy) in 2001 on alfalfa, and periodically refreshed. Rearing conditions were: 20 ± 1 °C, 18 h light/6 h dark, 65 ± 5% RH (see [66] for more details). A Perspex four-arm olfactometer [67] lit from above by diffuse, uniform lighting and maintained at 23 °C was used in a first set of tests. The bottom of the apparatus was lined with filter paper (110 cm diameter, Whatman), and air was removed through the four arms toward the center at 400 mL min^−1^. Single female parasitoids were introduced into the central chamber, and the time spent and number of entries into each arm were recorded by using specialist software (OLFA; Exeter Software, Setauket, NY, USA) over a 16-min period. The apparatus was rotated of one-quarter turn every 2 min to eliminate any directional bias. Aliquots (1 µL, extracted with diethyl ether, see Section 4.4) of VOC emitted by dSST and control plants were applied to a filter paper strip, and the solvent was allowed to evaporate for 30 s. The filter paper was then placed at the end of one arm. The three control arms were similarly treated with 1 µL of redistilled diethyl ether alone on filter paper. For each odor source at least 8 females were tested in three different days. The average time spent and the number of entries in treated or untreated arms was calculated. Data were then compared by one-sample t test. For wind tunnel bioassay 4-week old plants were used. For each line, a total of ten plants was used and offered individually daily in a random order to reduce any bias related to the time of the experiments. One hundred parasitoid females were tested singly for each target in no-choice experiments, and observed for a maximum of 5 min. The percentage of response (oriented flights, landings on the target) to each target plant was calculated. Resulting values were compared by a G-test of independence [68]. The parameters of the bioassay were set as follows: temperature, 20 ± 1 °C; 65 ± 5% RH; wind speed, 25 ± 5 cm s^−1^; distance between releasing vial and target, 50 cm; PPFD at releasing point, 700 μmol m^2^ s^−1^.

### 4.4. VOC Collection and Analysis

Volatiles from 4 weeks-old dSST plants and relative control, with at least four expanded leaves (*e.g.*, Figure S1) were collected by an air-tight entrainment system consisting of a glass jar (20 dm^3^) connected to a circulating pump (closed-loop) whose flow was adjusted to 200 cm^3^ min^−1^. Before re-entering the pump, the air passed through a glass narrow tube filled with a biphasic phase of 30 mg of Tenax and 20 mg of Carboxen (GERSTEL GmbH & Co.KG, Mulheim an der Ruhr, Germany). Glass jars, cleaned using diethyl ether, and pipelines were used on each measurement, to avoid memory effects. Ten plants for each genotype, were placed singly inside glass jars and VOCs were collected from the system for 3 h (totalling 3.6 dm^3^ of air sampled) under PPFD of 700 μmol m^2^ s^−1^, temperature of 25 ± 2 °C and RH of 50 ± 10% in order not to cause anomalous plant responses caused by simultaneous un-controlled decrease in [CO_2_] and increase in RH inside the glass jar. All VOCs were eluted from tube with 50 μL of redistilled diethyl ether. An Agilent 7890 GC-chromatograph coupled with an Agilent 5975C MSD spectrometer (Agilent Technologies, Santa Clara, CA, EUA) was used to analyse VOCs [69]. The following chromatographic conditions were used: column HP-Innovax polyethylene glyco (50 m, 200 μm, ID 0.4 μm DF); splitless mode, oven programme: 40° for 1 min, then a 5 °C min^−1^ ramp to 200 °C, a 10 °C min^−1^ ramp to 220 °C, and a 30 °C min^−1^ ramp to 260 °C, final temperature held for 3.6 min. Mass spectra were acquired within the 29–350 m/z interval operating the spectrometer at 70 eV and at scan speed mode. Three scans s^−1^ were obtained. The identification of VOCs was done on the basis of both matches of the peak spectra with library spectral database, and comparison with pure standards (Appendix A). All standards were purchased by Sigma-Aldrich (Milan, Italy). After identification, each VOC found in the samples was quantified through regression lines built by using a set of serial dilutions of pure standards covering similar spans of VOCs as in sampled leaves. Data were analysed using Agilent MassHunter Workstation software (Agilent 7890A; Agilent Agilent Technologies, Santa Clara, CA, USA). The volatile emission patterns, measured as peak areas divided by fresh plant weight, were analysed by PLS-DA (Partial least squares Discriminant Analysis) and Anova followed by Tukey’s or pairwise Kruskal-Wallis test (*p* < 0.05), according to data distribution. Before PLS-DA, data were square root transformed mean-centred and scaled to unit variance using the “ropls” R package [70].

### 4.5. Microarray and Functional Analysis

Two transgenic lines (dSST26 and 33) and untransformed control ‘Red Setter’ were analysed by microarray in three biological replicates. Leaf tissues from 4 weeks-old seedlings were powdered in liquid nitrogen and homogenised in Qiazol solution (Qiagen, Milan, Italy). Total RNA was extracted using Plant RNeasy mini kit (Qiagen) according to manufacturer’s protocol. RNA samples were analysed quantitatively and qualitatively by NanoDrop ND-1000 Spectrophotometer (NanoDrop Technologies, Berlin, Germany) and by Bioanalyser (Agilent Technologies, Santa Clara, CA, USA). Only samples with 260/280 nm absorbance ≥ 1.8 and 260/230 nm absorbance ≥ 2, were used for RNA labelling. Total RNA from transgenic and control samples was amplified in the presence of cyanine-3/cyanine-5 labelled CTP using Agilent low RNA Input Agilent’s Quick Amp Labeling kit for two-color (Agilent Technologies, Santa Clara, CA, USA) according to manufacturer’s protocol. All samples were processed together with Agilent’s RNA spike kit. After labelling, samples were purified using RNeasy mini spin column (Qiagen, Milan, Italy) to remove unincorporated dye-labelled nucleotides. The quality of labelled targets was determined by calculating the amount of cDNA produced, the pmoles of dye incorporated and the frequency of incorporation by NanoDrop. The specific activity was calculated using the formula: (Concentration of Cy3 or Cy5)/(Concentration of cRNA) * 1000 = pmol Cy3 or Cy5 per μgcRNA.Equal amounts of cRNAs (825 ng) from control (labelled with Cy3) and from transgenic samples (labelled with Cy5) were mixed together and hybridized to the Agilent’s 4 × 44k tomato chips in a hybridization oven at 65 °C for 17 hours with rotation at 10 rpm. After hybridization slides were washed with Gene Expression Wash buffer 1 for 1 min at room temperature and Gene Expression Wash buffer 2 for 1 min at 37 °C. Finally, to dry the slides and prevent ozone degradation arrays were treated with the Stabilization and Drying Solution (Agilent Technologies) for 30 s at room temperature. After washing, slides were scanned with the Agilent’s dual laser microarray scanner (G2565AA) and image data were processed using Agilent Feature extraction software (FE) (Agilent Technologies, Santa Clara, CA, USA). This software calculates log ratios and p-values for valid features on each array and provides a confidence measure of gene differential expression performing outlier removal and background subtraction. The raw data and associated sample information were loaded and processed by GeneSpring GX 10 (Agilent Technologies, Santa Clara, CA, USA). Statistical analysis was performed using background-corrected mean signal intensities from each dye channel. Microarray data were normalized using intensity-dependent global normalization (LOWESS). Differentially expressed RNAs were identified using a filtering by the Benjamini and Hochberg False Discovery Rate (*p*-value < 0.05) to minimize selection of false positives. Of the significantly differentially expressed RNA, only those with greater than 2-fold increase or 2-fold decrease in expression compared to the controls were used for further analysis. a similarity analysis was conducted with blastN against SGN Tomato Unigene database (http://solgenomics.net/). We used an e-value threshold of 1e-10 to reduce redundancy on the array as well as possible imperfect probe matches. The comparative analysis was performed against a combination of all Tomato Unigenes, BACs, and BAC-end sequences predicted by the ITAG (International Tomato Annotation Group) official annotations, on the SL2.40 tomato genome build. Briefly, when possible, we located each probe on the tomato genome and associated each probe to a transcript of a single gene. Microarray results were verified by real time RT-PCR targeting 14 genes indicated in Table 2. cDNA was synthetized on the same RNA samples used for the microarray analysis and Real Time RT-PCR was performed as described above.

The Agilent’s 4 × 44k tomato array (43803 probes) is based on known tomato genes, but also on annotated ESTs and cDNA sequence information. Differentially expressed sequences found in this study were re-annotated using different methods to get a proper functional annotation for the unknown gene names. Fasta format sequences were downloaded from NCBI, The Tomato Gene Index (DFCI) and Plant Transcript Assemblies Database (TIGR). Functional annotation and data mining were performed using Blast2GO software (CIPF, Valencia; www.blast2go.org). The analysis carried out with Blast2GO was performed using the software default parameters. It started with a Blastx similarity search against the *nr*NCBI protein database in order to collect a hit list. Statistically significant matches were then assigned to each query, and GO annotations were mapped from known associations. The analysis was enriched with InterProScan and Annex tools available in Blast2GO. GOslim tool helped to create simpler ontological categories and Kegg pathway analysis to underline biological pathways involving the submitted sequences.

### 4.6. Direct Defence Biossay

The level of direct defences was assessed by a feeding bioassay with *Spodoptera exigua*(Lepidoptera: Noctuidae) moth. Larvae were grown in an environmental chamber at 25 ± 2 °C, 70 ± 5% RH and fed with an artificial diet composed by 41.4 g L^−1^ wheat germ, 59.2 g L^−1^ brewer’s yeast and 165 g L^−1^ corn meal, supplemented with 5.9 g L^−1^ ascorbic acid, 1.8 g L^−1^ methyl 4-hydroxybenzoate and 29.6 g L^−1^ agar. About 30 eggs hatched on this artificial diet and allowed to grow until the second instar. Uniform second instar larvae, were selected and separated in three groups of 10–12 members and each group was used to evaluate larval weight after feeding on dSST plants and relative control leaf disks. Single larvae were isolated in a tray well (Bio-Ba-8, Color-Dec, Italy) covered by perforated plastic lids (Bio-Cv-1, Color-Dec Italy), containing 2% agar (w/v) to create a moist environment required to keep turgid the experimental tomato leaf disks. Larvae were offered leaf disks of uniform size daily, initially of 2 cm^2^ and later of larger dimensions (3 to 5 cm^2^) following larval growth. Plastic trays were kept at 28 °C 16:8 h light/dark photoperiod (see [71] for more details). Larval weight and mortality were recorded until pupae development and analysed by Fisher's least significant difference.

## Figures and Tables

**Table 1:** Volatile organic compounds emitted by transgenic (dSST26 and 33) and relative control (Red Setter) plants (*n* = 10). Values are means ± standard error. Significative differences of compounds emission between both transgenic lines and control plant are bolded (Anova or Kruskal-Wallis test; *p* < 0.05).

		Mean Value of Volatiles Organic Compounds VOC (ng mg^−1^ fr wt) ± SE		
Compound Groups	Compound	dSST26	dSST33	Red Setter
Norisoprenes	2,4 dimethyl 1 heptene	8.21 ± 1.32	8.32 ± 1.35	8.27 ± 1.55
	6 methyl 5 hepten 2 one	0.04 ± 0.03	0.13 ± 0.11	0.15 ± 0.08
		8.25 ± 1.35	8.45 ± 1.46	8.42 ± 1.63
Aldehydes	nonanal	0.33 ± 0.08	0.39 ± 0.05	0.47 ± 0.07
Monoterpenes	linalool	0.27 ± 0.02	0.22 ± 0.03	0.3 ± 0.04
	α pinene	0.19 ± 0.01	0.17 ± 0.02	0.21 ± 0.03
	limonene	0.09 ± 0.01	0.07 ± 0.01	0.08 ± 0.01
	β phellandrene	0.12 ± 0.03	0.08 ± 0.02	0.13 ± 0.02
	p cymene	0.1 ± 0.04	0.07 ± 0.04	0.01 ± 0.01
	camphor	0.06 ± 0.03	0.07 ± 0.03	0.05 ± 0.03
		0.83 ± 0.14	0.68 ± 0.15	0.78 ± 0.14
Hydrocarbons	**dodecene**	**1.02 ± 0.41**	**1.31 ± 0.37**	**2.32 ± 0.37**
	4 methyl nonane	0.4 ± 0.14	0.62 ± 0.17	0.51 ± 0.17
		1.42 ± 0.55	1.93 ± 0.54	2.83 ± 0.54
Alcohols	2 ethyl 1 hexanol	0.84 ± 0.4	1.05 ± 0.36	1.04 ± 0.28
	benzil alcol	0.05 ± 0.05	0.31 ± 0.13	0.07 ± 0.07
		0.89 ± 0.45	1.36 ± 0.49	1.11 ± 0.35
Benzenoids	ethylbenzene	0.41 ± 0.06	0.61 ± 0.14	0.52 ± 0.1
	**benzaldehyde**	**0.12 ± 0.08**	**0.26 ± 0.11**	**0.61 ± 0.22**
	4 methyl benzaldehyde	3.6 ± 0.81	3.36 ± 0.81	2.01 ± 0.46
	**2,4 dimethyl benzaldehyde**	**0 ± 0**	**0.01 ± 0.01**	**0.05 ± 0.03**
	2,5-dimethyl benzaldehyde	0 ± 0	0.06 ± 0.04	0 ± 0
	**trimethyl benzene**	**0.09 ± 0.06**	**0.13 ± 0.06**	**0.26 ± 0.1**
	1,4 dichlorobenzene	0.29 ± 0.25	0.62 ± 0.38	0.16 ± 0.09
	benzothiazole	0.09 ± 0.04	0.12 ± 0.06	0.13 ± 0.05
	methyl benzoate	0.22 ± 0.1	0.38 ± 0.08	0.35 ± 0.07
	acetophenone	0.49 ± 0.12	0.47 ± 0.11	0.41 ± 0.06
	**naphthalene**	**2.52 ± 1.4**	**3.06 ± 1.3**	**1.5 ± 0.31**
	4 vinylphenol	0 ± 0	0 ± 0	0.01 ± 0.01
	2 phenoxyethanol	0.24 ± 0.17	0.04 ± 0.03	0.19 ± 0.11
	pXylene	1.66 ± 0.26	1.6 ± 0.31	1.98 ± 0.28
		9.73 ± 3.35	10.72 ± 3.44	8.18 ± 1.89
	Total VOC	21.45 ± 5.92	23.53 ± 6.13	21.79 ± 4.62
	Plant Weight (g)	2.43 ± 0.24	2.35 ± 0.29	2.50 ± 0.12

**Table 2:** Gene-specific primers used in real-time RT-PCR and relevant parameters.

Primers	Sequence 5′ to 3′	Ta (°C)	Lenght (base pairs)	Gene or Expressed Sequence Tag	National Center for Biotechnology Information
GCSFw GCSRw	TTGGTGAAGCCTTAACTCAGCC GCAAATGGTGGTGTGCATCAT	60	101	*GCS*	AF035630
PcEF1FwRt PcEF1RwRt	CTCCATTGGGTCGTTTTGCT GGTCACCTTGGCACCAGTTG	60	101	*EF1α*	X14449
PcFPSFwRt PcFPSRwRt	GCAAAGCAGTACAGGCAGTGC TCCCAATGGGAGAATGAAGTTC	60	101	*LeFPS1*	AF048747
TDC-F TDC-R	ACTGTTAGCTCCGCTGCGTTT CCATTTCCAACTCCGTGCAT	60	105	*TDC*	AA824781
ArgE-F ArgE-R	ACAACCCACCGGATCTTATCC TGATGATCAATGCTCCGCC	60	102	*ArgE*	AW737876
ACoC-F ACoC-R	CAAAGAGGCGGAAGTTCACAAA CCAAAGTTTCACCTCCCACTCA	60	114	*ACoC*	AW928749
ES895666–F ES895666-R	TACACAGTTTGGCCCGGTACA CAGGAGCGGAGAGTTGGATAGT	60	103	ES895666	ES895666
PAL-F PAL-R	GCTGAGCAACACAACCAAGATG TGGCAAAGAGCCACGAGATAG	60	116	*PAL*	M83314
BF097728-F BF097728-R	GGTTTTATGGTGCTGCTTCACT GCAAATCGGAAAAGCCCAC	60	172	BF097728	BF097728
RO-F RO-R	CAGAAGCTGTAATGGAGCCAGG GCAAAGTTCATGCCTTCCCAG	60	102	*RO*	BP881050
ACS-F ACS-R	CGACGAAATATACGCTGGCAC TGCCAACTCTGAAACCTGGAA	60	161	*ACS*	M83322
AY348872-F AY348872-R	TGCTGGTGCACGCTTTGTT CGCAGTAAGCCAATTCGTGAG	60	102	AY348872	AY348872
PXD-F PXD-R	CCCCGGCATTGTTTCTTGT GGCATCTCTTCTGCCCAATTTT	60	88	*PXD*	AK320453
F35H-F F35H-R	ACGTTCGTGCCAATGAGCTAG CCATCGCAAACGTTAACACATC	60	103	*F35H*	EU626067 GQ904194
ACS3-R ACS3-F	CGCAAAAAAGCGCAACCTT GTGAATGCCTTTTTCGTCGATG	60	109	*ACS3*	U17972.1

## Supplementary Materials

Supplementary Materials can be found at http://www.mdpi.com/1422-0067/19/9/2748/s1. Figure S1. Phenotype of the transgenic T1 lines dSST26-33 and relative un-transgenic cultivar “Red Setter” four weeks (a) and eight weeks after sowing (b). Figure S2. Array gene expression verification by real-time RT-PCR. (a) For each gene, the graph displays the relative quantity of the target in transformed plants (white columns) and untransformed control cv ‘Red Setter’ (black columns). Quantities (RQ) are shown relative to the calibrator ‘Red Setter’ genotype. The list of the target genes analyzed is reported on the x-axis; the results of the relative quantification calculations are reported on the y-axis. (b) Points represent fold changes (log^2^RQ) for each gene common between array and real time RT-PCR. The continuous line on graph represents linear regression. Table S1. Differentially expressed genes annotation. The automatic BLAST annotation of sequences was performed by the BLAST2GO software suite and the fold change of transgenic plant compared to relative control cv Red Setter, expectation value (e value), sequence similarity and corresponding Gene Ontology terms were reported (---NA--- represents sequences not annotated, in bold were reported sequence validated by realtime RT-PCR—see Figure S2—in brackets we reported the name of the genes used in the paper).

## Appendix A. Standards Used for the Identification of Volatiles Collected by Air-Entrainment of Head Space from Tomato Plants

(+) longifolene, (Z)-3-hexen-1-ol, 3-carene, 6-methyl- 5-hepten-2-one, anisole-p-allyl, camphor, chlorobenzene, cis-nerolidol, decane, dodecene, eucalyptol, eugenol, hexanal, humulene (=α-caryophyllene), linalool, methyl salicilate, menthol, ocimene, p-cymene, p-dichlorobenzene (IS), phellandrene, R(+) limonene, S(-) limonene, skatol, terpinolene, (E)-ß-caryophyllene, trans-nerolidol, trans-ß-farnesene, α-copaene, α/cubebene, α-gurjunene, α-pinene, α-terpinene, α-terpineol, ß-myrcene, γ-terpinene.
