# Supplementary material for: TPS Genes Silencing Alters Constitutive Indirect and Direct Defense in Tomato
Source: Int J Mol Sci. 2018 Sep 13;19(9):2748. doi: 10.3390/ijms19092748 (PMC6163538; doi:10.3390/ijms19092748)
Supplement: Supplementary file 1 [file ijms-19-02748-s001.pdf]

Supplementary Materials:

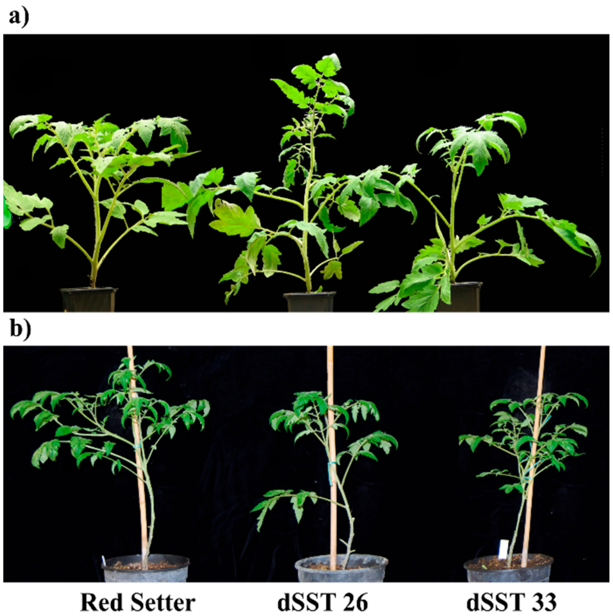

Figure S1. Phenotype of the transgenic T1 lines dSST26-33 and relative un-transgenic cultivar "Red Setter" four weeks (**a**) and eight weeks after sowing (**b**).

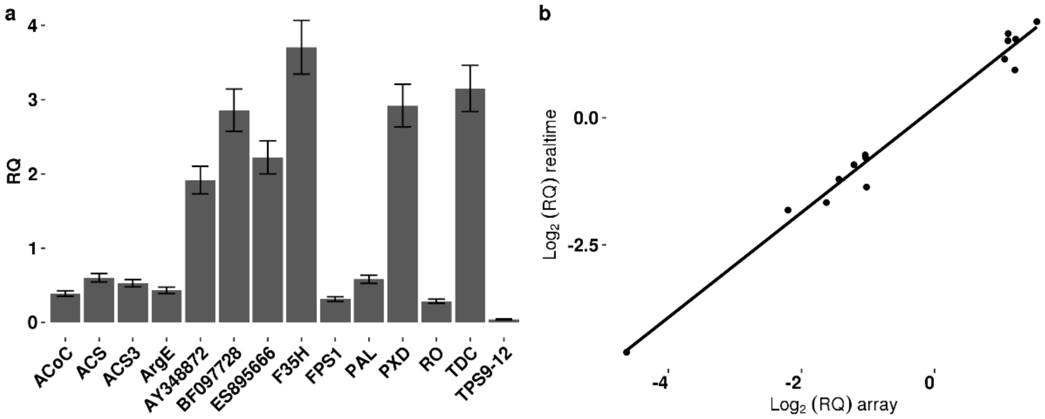

Figure S2. Array gene expression verification by real-time RT-PCR. (**a**) For each gene, the graph displays the relative quantity of the target in transformed plants (white columns) and untransformed control cv 'Red Setter' (black columns). Quantities (RQ) are shown relative to the calibrator 'Red Setter' genotype. The list of the target genes analyzed is reported on the x-axis; the results of the relative quantification calculations are reported on the y-axis. (**b**) Points represent fold changes (log²RQ) for each gene common between array and real time RT-PCR. The continuous line on graph represents linear regression.

mRNA/EST ID	UniGene ID	Fold Change	Gene Ontology Annotation	GO terms	Min. eValue	Mean Similarity
TC197491	SGN-U579809	2.447	Cytochrome p450	GO:0009793; GO:0009086; GO:0019745; GO:0000394; GO:0016126	6.31E-153	79.45%
**U17972.1 (ACS3)**	**SGN-U565891**	**-2.317**	**1-aminocyclopropane-1-carboxylate synthase**	GO:0071281; GO:0009693; GO:0009733; GO:0005737; GO:0016847; GO:0042802; GO:0005515	**0**	**90.00%**
TA36168_4081	SGN-U581138	2.634	24-sterol c-methyltransferase	GO:0006084; GO:0016132; GO:0042398; GO:0009805; GO:0009793; GO:0019745; GO:0009698; GO:0006598; GO:0009611; GO:0016126; GO:0005737; GO:0003838	3.49E-73	87.10%
DB707884.1	SGN-U563783	-2.075	beta-ketoacyl- synthase	GO:0006633	1.49E-42	67.00%
**AW928749.1 (AcoC)**	**SGN-U601457**	**-2.033**	**Acetyl-CoA Carboxylase**	GO:0009507; GO:0005737; GO:0003989	**1.72E-68**	**76.55%**
**AW737876.1 (ArgE)**	**SGN-U591649**	**-2.711**	**acetylornithine deacetylase**	GO:0006526; GO:0008652	**8.46E-107**	**84.55%**
**TA40884_4081 (TPS9-11)**	**SGN-U580988**	**-25.027**	**sesquiterpene synthase**	GO:0051762; GO:0033383	**4.75E-117**	**73.90%**
AK327963.1	SGN-U576202	2.357	Dolichol kinase	GO:0008150	0	75.95%
TA54084_4081	SGN-U566374	-2.186	ap2 erf domain-containing transcription factor	GO:0009873, GO:0003700, GO:0009753, GO:0009861, GO:0005667, GO:0045449	4.51E-65	76.40%

mRNA/EST ID	UniGene ID	Fold Change	Gene Ontology Annotation	GO terms	Min. eValue	Mean Similarity
**AK328022.1 (F35H)**	**SGN-U602093**	**2.896**	**flavonoid 3’ 5’ -hydroxylase**	GO:0009718; GO:0016711	**0**	**94.65%**
AK320815.1	SGN-U580204	-2.217	dna repair protein rad23-3-like	GO:0007267; GO:0000911; GO:0006281; GO:0006302; GO:0000226; GO:0035196; GO:0006396	2.31E-115	85.35%
BW692890	SGN-U581282	2.161	hsp22	GO:0006457; GO:0009408; GO:0009644; GO:0042542; GO:0009507	1.21E-77	75.15%
ES894405.1	SGN-U570087	-2.161	benzoic acid/salicylic acid methyltransferase	GO:0005739	1.37E-76	78.45%
DB723477.1	SGN-U570573	-2.033	malic enzyme	GO:0005739	4.97E-83	84.95%
CD003371.1	SGN-U597254	-2.157	uncharacterized protein	---NA---	1.96E-62	84.50%
AK321037.1	SGN-U574780	2.702	delta-12 fatty acid desaturase	GO:0046471, GO:0005575, GO:0080167, GO:0006636, GO:0009507, GO:0052637	0	79.95%
AK325335.1	SGN-U574560	-3.207	pectinesterase family protein	GO:0016023	0	66.70%
U50152.1	SGN-U577971	-2.404	leucine aminopeptidase	GO:0002213, GO:0009507	0	88.45%
**AK320453.1 (PXD)**	**SGN-U580709**	**2.323**	**peroxidase**	GO:0002215, GO:0055114, GO:0004601, GO:0020037	**0**	**88.65%**
**TC199251 (PAL)**	**SGN-U577677**	**-2.052**	**phenylalanine ammonia-lyase**	GO:0005737, GO:0045548, GO:0009698, GO:0006559, GO:0006570, GO:0009821	**0**	**92.85%**
AK319177.1	SGN-U574365	2.832	serine threonine protein phosphatase 2a 57 kda regulatory subunit b iota isoform-like	GO:0005515, GO:0005618, GO:0050832, GO:0006952, GO:0007165	0	84.25%

mRNA/EST ID	UniGene ID	Fold Change	Gene Ontology Annotation	GO terms	Min. eValue	Mean Similarity
TC210229	SGN-U580850	2.329	Pyruvate kinase	GO:0016310, GO:0009507		
TC215254		-2.138	hypothetical protein VITISV_024659 [Vitis vinifera]	---NA---	6.69E-63	82.00%
BI931808.1	SGN-U584472	-2.155	regulatory-associated protein of tor 1-like	---NA---	2.12E-139	87.85%
TC192744	SGN-U579243	2.173	60s ribosomal protein l10a	GO:0022625, GO:0005622, GO:0005840, GO:0006412, GO:0003735, GO:0042254, GO:0005737	1.92E-121	94.05%
**AA824781 (TDC)**	**SGN-U598816**	**2.148**	**tryptophan decarboxylase**	GO:0052544; GO:0042742; GO:0050832; GO:0016045; GO:0009595; GO:0009867; GO:0000165; GO:0010310	**3.29E-70**	**84.95%**
TC192232	SGN-U573178	3.796	ubiquitin-activating enzyme	GO:0006511, GO:0004842	1.13E-127	89.65%
BI210771	SGN-U593459	2.028	ubiquinol--cytochrome-c reductase - like protein	GO:0005739	6.04E-38	89.90%
TC197326		-17.556	cysteine protease inhibitor	GO:0012505, GO:0008289, GO:0006869, GO:0006810	4.55E-150	93.50%
AW219265.1	SGN-U591461	-4.909	uncharacterized protein	---NA---	3.18E-11	58.13%
**BP881050.1 (RO)**	**SGN-U597634**	**-4.604**	**Reticulin oxidase**	GO:0055074; GO:0006457; GO:0046686; GO:0009408; GO:0042542; GO:0006979; GO:0009651	**2.35E-22**	**82.40%**
TC208821	SGN-U568521	-3.999	long-chain-alcohol oxidase fao1-like	GO:0006066; GO:0055114; GO:0043231; GO:0009055; GO:0046577	8.98E-70	69.70%

mRNA/EST ID	UniGene ID	Fold Change	Gene Ontology Annotation	GO terms	Min. eValue	Mean Similarity
AW737824.1	SGN-U597233	-3.818	2pgk 2-phosphoglycerate kinase	GO:0006096; GO:0046685; GO:0046686; GO:0034976; GO:0048046; GO:0009570	1.47E-36	87.75%
BG134534.1	SGN-U579280	-3.811	formate dehydrogenase	GO:0046686, GO:0009611, GO:0009507, GO:0005739, GO:0016616	3.29E-10	60.20%
AK327523.1	SGN-U583362	-3.735	s-locus linked f-box protein type-3	GO:0005515, GO:0005488	2.28E-48	46.60%
AK246698.1	SGN-U592051	-3.529	maf-like protein	---NA---	1.11E-92	80.20%
**AK324941.1 (FPS)**	**SGN-U580757**	**-3.089**	**farnesyl diphosphate synthase**	GO:0006084; GO:0016132; GO:0006816; GO:0030003; GO:0042398; GO:0045337; GO:0009698	**0**	**89.40%**
BT014156.1	SGN-U583400	-3.262	---NA---	---NA---		
BF050158.1	SGN-U573768	-3.235	rna-dependent rna polymerase family protein	GO:0003968, GO:0016441, GO:0016740, GO:0050789	1.77E-28	58.60%
TC214521	SGN-U584654	-3.213	protein cobra-like	GO:0016023	1.99E-153	90.45%
TC213108	SGN-U563907	-3.135	seed storage protein vicilin partial	---NA---	1.46E-91	64.25%
AW622775.1	SGN-U598495	-3.132	predicted protein [Populus trichocarpa]	---NA---	6.54E-06	53.00%
AW036162.1	SGN-U597479	-3.062	---NA---	---NA---		
BE431535.1	SGN-U601336	-2.921	Porin autotransporter (AT) family	GO:0019344, GO:0009086, GO:0010043, GO:0000394, GO:0005737, GO:0005783, GO:0005794, GO:0005773, GO:0004046	1.36E-25	93.40%

mRNA/EST ID	UniGene ID	Fold Change	Gene Ontology Annotation	GO terms	Min. eValue	Mean Similarity
BG133638.1	SGN-U579572	-2.917	pathogenesis-related protein STH-2	GO:0006355, GO:0005634, GO:0005515, GO:0003700, GO:0008270		
AW933519.1	SGN-U598660	-2.728	pentatricopeptide repeat-containing protein	GO:0003674, GO:0005575, GO:0008150	1.25E-04	64.00%
AK328086.1	SGN-U602225	-2.692	F-box family protein	GO:0005739	4.27E-163	73.70%
BI209303.1		-2.650	gag-pol polyprotein	GO:0006310; GO:0006278; GO:0003677; GO:0004523;	3.19E-101	62.95%
BP908250.1	SGN-U597898	-2.629	myosin subfamily xi heavy chain	GO:0012505, GO:0016987, GO:0017111, GO:0051301, GO:0051645, GO:0051646, GO:0005524	1.76E-68	89.35%
AK322282.1	SGN-U568388	-2.607	---NA---	---NA---		
DB684494.1	SGN-U603706	-2.596	transcription factor tcp14-like	GO:0030154; GO:0009793; GO:0016226; GO:0048366; GO:0009965; GO:0009657	1.52E-125	70.25%
BP907314.1	SGN-U564379	-2.560	2-oxo acid lipoyl-binding site	GO:0005739	5.84E-68	76.95%
AW624572.1	SGN-U577570	-2.559	Ribulose 1,5-bifosphate carboxylase/oxygenase activase 2	GO:0015977, GO:0009573, GO:0009536		
TC215043	SGN-U580407	-2.554	ALF5 aberrant lateral root formation 5 antiporter	---NA---		
TC216107	SGN-U574155	-2.525	glycine-rich rna-binding protein	GO:0045087, GO:0009737, GO:0009409, GO:0009651, GO:0010043, GO:0005618, GO:0003676	3.90E-36	77.70%
BP907391.1	SGN-U572701	-2.515	AF378084_1PNCBP [Solanum tuberosum]	---NA---	3.43E-73	82.00%

mRNA/EST ID	UniGene ID	Fold Change	Gene Ontology Annotation	GO terms	Min. eValue	Mean Similarity
BP876774.1	SGN-U597637	-2.494	abscisic insensitive 1b	GO:0051301, GO:0048825, GO:0009790, GO:0009740, GO:0009686, GO:0031930, GO:0009657, GO:0045893, GO:0006355	1.10E-23	62.30%
AK327977.1	SGN-U578327	-2.485	cbs domain protein	GO:0045454, GO:0006096, GO:0007030, GO:0006972, GO:0046686, GO:0009651, GO:0009266, GO:0006833	2.49E-41	74.55%
AA824688.1	SGN-U604678	-2.470	chromatin remodeling complex subunit	GO:0006338, GO:0048573, GO:0040029, GO:0005634, GO:0016514, GO:0003677, GO:0005515	4.67E-58	100.00%
AK246590.1	SGN-U578028	-2.420	glycine-rich rna-binding protein 2	GO:0045087, GO:0009737, GO:0009409, GO:0009651, GO:0010043, GO:0005618, GO:0003676	1.01E-78	83.00%
TC211674	SGN-U566586	-2.415	Zinc ion binding	GO:0008270, GO:0055114	1.97E-18	47.60%
BI210263.1	SGN-U602079	-2.398	ABI2 ABA insensitive 2 protein serine/threonine phpsphatase	GO:0010152, GO:0006355, GO:0009737, GO:0009409, GO:0009651, GO:0009414, GO:0005634		

mRNA/EST ID	UniGene ID	Fold Change	Gene Ontology Annotation	GO terms	Min. eValue	Mean Similarity
CD002235.1	SGN-U600207	-2.365	atpase splayed	GO:0015991, GO:0006486, GO:0015992, GO:0010119, GO:0009737, GO:0009414, GO:0005794, GO:0016021	4.96E-18	88.95%
DB706064.1	SGN-U579949	-2.361	Cellulose synthase-like protein G3	GO:0016740, GO:0016020, GO:0016757, GO:0016759, GO:0016760, GO:0043170	1.43E-88	78.45%
BG629748.1	SGN-U584382	-2.361	spc97 spc98 family of spindle pole body component	GO:0007020, GO:0000930, GO:0009898, GO:0005635, GO:0015631	1.22E-19	62.64%
TC193947		-2.355	er auxin binding protein 1 precursor	GO:0000910, GO:0051781, GO:0045793, GO:0032877, GO:0009826, GO:0010011	5.32E-15	90.00%
TC209356	SGN-U581798	-2.347	short-chain dehydrogenase/reductase SDR family protein	GO:0047501, GO:0005886, GO:0005829, GO:0009744, GO:0080167, GO:0055114, GO:0009813, GO:0006952	1.49E-78	69.20%
BE432867.1	SGN-U570176	-2.340	---NA---			
TC212535	SGN-U579923	-2.332	e3 ubiquitin-protein ligase ubr4	GO:0006511, GO:0004842	7.49E-107	81.85%
BG630161.1	SGN-U604261	-2.332	---NA---	---NA---		
AW615915.1	SGN-U603473	-2.321	potyviral helper component protease-interacting protein 1	GO:0005739	3.79E-76	66.40%
AK247833.1	SGN-U576138	-2.315	uncharacterized protein	---NA---	4.39E-44	59.00%
AW443638.1	SGN-U598572	-2.302	beta-glucosidase 44-like	GO:0005618, GO:0005975, GO:0004565, GO:0009505	5.14E-15	42.88%

mRNA/EST ID	UniGene ID	Fold Change	Gene Ontology Annotation	GO terms	Min. eValue	Mean Similarity
BT013974.1	SGN-U570151	-2.298	rna recognition motif-containing protein	GO:0005739	0	79.55%
AK323609.1	SGN-U570482	-2.295	Kinesin heavy chain isolog	GO:0055046, GO:0048451, GO:0000914, GO:0080175, GO:0048453, GO:0009524, GO:0003777, GO:0008574	3.16E-81	66.85%
BE354788.1	SGN-U578389	-2.293	kunitz-type protease inhibitor precursor	GO:0050832, GO:0009620, GO:0009751, GO:0004867	5.62E-51	65.85%
AI898032.1	SGN-U563285	-2.279	homeodomain-leucine zipper protein	GO:0006355, GO:0005634, GO:0003677	4.87E-65	78.35%
TC216008	SGN-U574197	-2.276	o-acyltransferase (wsd1-like) family protein	GO:0010025, GO:0005783, GO:0004144, GO:0047196	1.15E-50	63.05%
TC210351	SGN-U586176	-2.274	uncharacterized protein	---NA---	4.07E-30	49.06%
BI207234.1	SGN-U599925	-2.251	Zinc finger CCCH domain-containing protein 24	GO:0009793, GO:0006355, GO:0005634, GO:0003676, GO:0003700		
BG626093.1	SGN-U571622	-2.245	lysine histidine transporter	GO:0043090, GO:0006865, GO:0015696 , GO:0006820, GO:0015802, GO:0030968, GO:0006888, GO:0006499, GO:0043069, GO:0006862, GO:0006612, GO:0043269	2.56E-25	83.15%
DB694047.1	SGN-U574293	-2.232	Nucleic acid binding	GO:0006355, GO:0003677	2.05E-59	80.75%

mRNA/EST ID	UniGene ID	Fold Change	Gene Ontology Annotation	GO terms	Min. eValue	Mean Similarity
TA53846_4081	SGN-U571987	-2.225	Ankyrin repeat-domain containing protein 2	GO:0042742, GO:0050832, GO:0045087, GO:0009867, GO:0000165, GO:0031348, GO:0043069, GO:0006612, GO:0010310, GO:0045088, GO:0010363, GO:0009751, GO:0009697, GO:0009862		
BF097872.1	SGN-U565631	-2.220	chromatin remodeling complex subunit	GO:0006338, GO:0048573, GO:0040029, GO:0005634, GO:0016514, GO:0003677, GO:0005515	4.87E-84	98.40%
FS197580.1	SGN-U601965	-2.220	dynamin-like protein	GO:0072583, GO:0009737, GO:0009504, GO:0005737, GO:0005829, GO:0005886	4.10E-32	87.80%
DB691535.1	SGN-U602213	-2.218	protein binding	GO:0005515	2.68E-36	51.05%
AW621582.1	SGN-U586258	-2.215	myosin heavy chain-like protein	GO:0012505, GO:0016987, GO:0017111, GO:0051301, GO:0051645, GO:0051646, GO:0005524	3.72E-36	60.30%
TC213299	SGN-U574928	-2.210	serine threonine-protein kinase nek5-like	GO:0012505, GO:0004672, GO:0010204, GO:0010359, GO:0005515, GO:0005524, GO:0004674, GO:0009626, GO:0016301	0	70.45%
AK325646.1	SGN-U579091	-2.189	Legumin type B	---NA---	0	66.65%

mRNA/EST ID	UniGene ID	Fold Change	Gene Ontology Annotation	GO terms	Min. eValue	Mean Similarity
TC191894	SGN-U572061	-2.188	Embryo defective 1507 ATP binding	---NA---		
TC204487		-2.182	proteinase inhibitor ii	GO:0009737, GO:0009607, GO:0009651, GO:0009611	5.33E-96	88.15%
AK324068.1	SGN-U570419	-2.159	uncharacterized protein	---NA---	4.56E-107	70.45%
AJ784473.1	SGN-U599708	-2.154	Zinc finger C3HC4 family protein	GO:0010200, GO:0005759, GO:0008270, GO:0009737		
TA38641_4081	SGN-U578022	-2.148	eukaryotic translation initiation factor 3	GO:0005737, GO:0005829, GO:0005852, GO:0005634, GO:0003676, GO:0003743	2.06E-48	71.90%
DY523815.1	SGN-U599172	-2.146	DMR6 Downy Mildew Resistant 6 oxidoreductase	GO:0042742, GO:0050832, GO:0009595, GO:0009813, GO:0045087, GO:0009867, GO:0000165, GO:0031348, GO:0006612, GO:0031347, GO:0010310, GO:0043900, GO:0010363, GO:0009617	1.02E-25	65.00%
DV105496.1	SGN-U568689	-2.144	pumilio homolog 2-like	GO:0009507	2.14E-27	66.20%
BM411846.1	SGN-U575359	-2.142	microtubule-associated protein	GO:0009653, GO:0051301, GO:0000902, GO:0048869, GO:0010106, GO:0043622, GO:0006826, GO:0010150, GO:0031115, GO:0015706, GO:0009737, GO:0009409, GO:0010167, GO:0010038, GO:0009739	1.04E-12	52.13%

mRNA/EST ID	UniGene ID	Fold Change	Gene Ontology Annotation	GO terms	Min. eValue	Mean Similarity
BT013019.1	SGN-U575406	-2.137	protein seuss-like 2	GO:0009790, GO:0048467, GO:0048481, GO:0005634	0	81.30%
BG627845.1	SGN-U565937	-2.136	---NA---	---NA---		
TC212033	SGN-U586258	-2.130	centromeric protein	---NA---	5.78E-22	58.00%
TC207785	SGN-U575481	-2.126	serine threonine-protein kinase	GO:0012505, GO:0004672, GO:0010204, GO:0010359, GO:0005515, GO:0005524, GO:0004674, GO:0009626, GO:0016301	5.63E-15	70.33%
AK247811.1	SGN-U598287	-2.115	ADL3 Arabidopsis dynamin-like 3 GTPase	GO:0072583, GO:0009737, GO:0009504, GO:0005737, GO:0005829, GO:0005886	2.73E-10	58.20%
AK325681.1	SGN-U582785	-2.112	rna-binding protein 5 10	GO:0009507, GO:0005737, GO:0005634, GO:0008143, GO:0003723	0	76.70%
BW686630.1	SGN-U592836	-2.096	---NA---	---NA---		
BG629468.1	SGN-U599643	-2.092	DNA replication ATP-dependent helicase	GO:0005634, GO:0008026		
AI490908.1	SGN-U570811	-2.090	o-acyltransferase wsd1-like	GO:0010025, GO:0005783, GO:0004144, GO:0047196	5.94E-60	69.95%
BI926635.1	SGN-U602785	-2.090	receptor protein	GO:0007165, GO:0005768, GO:0005794, GO:0005886, GO:0005802	2.42E-102	79.00%
AW031266.1	SGN-U594817	-2.088	uncharacterized protein	---NA---	3.73E-88	86.55%
TC210888	SGN-U571356	-2.082	---NA---	---NA---		

mRNA/EST ID	UniGene ID	Fold Change	Gene Ontology Annotation	GO terms	Min. eValue	Mean Similarity
TC210316	SGN-U585332	-2.081	aspartic proteinase-like protein	GO:0006096, GO:0007030, GO:0006972, GO:0048513, GO:0006623, GO:0006508, GO:0046686, GO:0009651, GO:0009266, GO:0006833	3.96E-65	68.30%
TA57069_4081	SGN-U567188	-2.075	unnamed protein product [Vitis vinifera]	---NA---	3.58E-06	50.67%
TC192564	SGN-U579906	-2.070	nbs-lrr resistance protein	GO:0006915, GO:0006952, GO:0005524	2.73E-15	70.18%
AK327773.1	SGN-U577583	-2.067	11-beta-hydroxysteroid dehydrogenase-like	GO:0009737, GO:0009741, GO:0008202, GO:0009507, GO:0070524, GO:0072582, GO:0072555, GO:0016491	6.89E-146	73.30%
BF112425.1	SGN-U597303	-2.066	translational activator gcn1	---NA---	8.62E-85	93.40%
CN641307.1	SGN-U596761	-2.064	mlo-like protein 6	GO:0005488, GO:0005507, GO:0005516, GO:0005622, GO:0005886, GO:0008219, GO:0030001, GO:0046872, GO:0006396, GO:0009817, GO:0016021	9.28E-74	89.65%
TC203712	SGN-U577254	-2.061	ubiquitin family protein	GO:0006511, GO:0004842	3.98E-36	58.60%
**M83322.1 (ACS)**	**SGN-U565891**	**-2.060**	**1-aminocyclopropane-1-carboxylate synthase**	**GO:0009693, GO:0030170, GO:0009835, GO:0016769, GO:0016847**	**4.01E-57**	**87.70%**

mRNA/EST ID	UniGene ID	Fold Change	Gene Ontology Annotation	GO terms	Min. eValue	Mean Similarity
AW929342.1	SGN-U577943	-2.056	myosin heavy chain	GO:0012505, GO:0016987, GO:0017111, GO:0051301, GO:0051645, GO:0051646, GO:0005524	1.32E-59	68.85%
AI777063.1	SGN-U568600	-2.055	serine threonine protein	GO:0012505, GO:0004672, GO:0010204, GO:0010359, GO:0005515, GO:0005524, GO:0004674, GO:0009626, GO:0016301	4.23E-99	92.10%
AW934352.1	SGN-U588534	-2.050	hydroxyproline-rich glycoprotein family protein	GO:0005488, GO:0005773, GO:0005886, GO:0005737	3.28E-12	75.31%
TC212621	SGN-U576896	-2.045	multidrug resistance-associated protein 6 (6) abc-	GO:0015700, GO:0046685, GO:0009407, GO:0005634, GO:0000325, GO:0009506, GO:0005774, GO:0005773, GO:0015446, GO:0042626, GO:0071992, GO:0005515	0	76.55%
DB717813.1	SGN-U567275	-2.044	Zinc ion binding	GO:0008270, GO:0055114	6.76E-21	81.30%

mRNA/EST ID	UniGene ID	Fold Change	Gene Ontology Annotation	GO terms	Min. eValue	Mean Similarity
BT013746.1	SGN-U570838	-2.038	atp binding	GO:0045010, GO:0043481, GO:0006200, GO:0010315, GO:0009926, GO:0010540, GO:0048440, GO:0007155, GO:0071555, GO:0009793, GO:0009640, GO:0009958, GO:0008361, GO:0009733, GO:0009637, GO:0009624	0	71.50%
AW979814.1	SGN-U572659	-2.031	1-phosphatydilinositol-4-phosphatase 5-kinase	GO:0042742, GO:0016310, GO:0007165, GO:0004721	5.75E-26	57.95%
TC202160	SGN-U586008	-2.028	probable histone-lysine n-methyltransferase atxr3-like	GO:0005783, GO:0005634, GO:0009506, GO:0016279	6.11E-109	77.45%
AW035871.1	SGN-U573555	-2.028	Lactoylglutathione lyase family protein	---NA---	1.23E-78	80.85%
BM411467.1	SGN-U564670	-2.027	peptidase a1 pepsin	---NA---	9.98E-48	50.00%
AK326757.1	SGN-U578297	-2.019	f-box protein pp2-b1	GO:0005515, GO:0005488	7.39E-43	58.75%
CD579120.1	SGN-U582906	-2.018	Dicarboxylate/Tricarboxylate carrier	GO:0009693, GO:0006839, GO:0010200, GO:0009612, GO:0009611, GO:0006810, GO:0005743, GO:0005310		
TC209247	SGN-U583451	-2.017	protein	---NA---	6.38E-30	52.80%
BP908346.1	SGN-U578022	-2.014	protein kiaa0664 homolog	---NA---	8.80E-52	60.74%
BI205578.1		-2.006	integrase core domain containing protein	GO:0006310, GO:0003677	1.16E-27	52.90%

mRNA/EST ID	UniGene ID	Fold Change	Gene Ontology Annotation	GO terms	Min. eValue	Mean Similarity
AK328092.1	SGN-U578448	-2.002	1-aminocyclopropane-1-carboxylate oxidase	GO:0046872, GO:0009693, GO:0006952, GO:0055114, GO:0009835, GO:0031418, GO:0009815	0	75.50%
FS198129.1	SGN-U583735	-2.001	multiple c2 and transmembrane domain-containing protein 2-like	GO:0008150, GO:0003674	2.14E-80	85.35%
DB679092	SGN-U596485	2.002	trna-dihydrouridine synthase a	---NA---	4.41E-36	79.40%
AW442644	SGN-U565915	2.013	protein	---NA---	4.14E-36	72.00%
FS205197	SGN-U603482	2.021	Two-component response regulator ARR17	GO:0009736, GO:0006355, GO:0005634, GO:0000156	8.45E-06	63.67%
DV104534	SGN-U592284	2.041	endomembrane protein 70	GO:0016023, GO:0016020	3.80E-33	86.85%
AK319943.1	SGN-U577548	2.044	crt homolog 1-like	GO:0008150, GO:0005634, GO:0005524	1.46E-159	87.90%
AI778436	SGN-U577946	2.047	histone h2b	GO:0008283, GO:0005730, GO:0005634, GO:0003677, GO:0005515	1.96E-48	98.70%
AK329954.1	SGN-U575325	2.057	udp-glucosyltransferase	GO:0009920, GO:0046482, GO:0010583, GO:0009751, GO:0009407, GO:0009507, GO:0009524, GO:0010294, GO:0005515, GO:0016757, GO:0080002	0	66.35%
**ES895666**	**SGN-U582479**	**2.071**	**pathogenesis-related protein 5-like**	GO:0009607, GO:0006952	**4.67E-109**	**77.15%**

mRNA/EST ID	UniGene ID	Fold Change	Gene Ontology Annotation	GO terms	Min. eValue	Mean Similarity
TC203234	SGN-U574784	2.071	polyphenol oxidase	GO:0046872, GO:0004097, GO:0009543, GO:0055114, GO:0006118, GO:0006570	3.63E-23	74.20%
AY359965.1	SGN-U573229	2.079	Receptor-like protein kinase	GO:0006468, GO:0005524, GO:0004674, GO:0004713	0	59.80%
AJ459817.1	SGN-U571098	2.086	inorganic pyrophosphatase 2-like	GO:0005768, GO:0005794, GO:0005886, GO:0005802, GO:0005773, GO:0009678	0	87.10%
AK247334.1	SGN-U586385	2.086	extensin-like protein	GO:0006869, GO:0012505, GO:0008289	1.49E-58	67.00%
BI421975	SGN-U583705	2.120	xenotropic and polytropic retrovirus receptor	GO:0016020	6.81E-10	69.50%
BI923172	SGN-U575858	2.131	cytochrome p450	GO:0016023, GO:0009055, GO:0020037, GO:0055114, GO:0019825, GO:0070330, GO:0006118	4.93E-68	72.15%
BP891736	SGN-U564623	2.133	protease inhibitor seed storage lipid transfer protein family protein	GO:0016023	3.65E-15	60.20%
BW691204	SGN-U577212	2.133	WRKY-like transcription factor	GO:0030010, GO:0009942, GO:0006355, GO:0005634, GO:0003700		
TC212704	SGN-U593710	2.136	cytochrome p450	GO:0016023, GO:0009055, GO:0020037, GO:0055114, GO:0019825, GO:0070330, GO:0006118	8.22E-67	85.15%

mRNA/EST ID	UniGene ID	Fold Change	Gene Ontology Annotation	GO terms	Min. eValue	Mean Similarity
**BF097728**	**SGN-U564327**	**2.144**	**disease resistance response protein 206-like**	GO:0008219, GO:0002229, GO:0000165, GO:0009788, GO:0046777, GO:0006487, GO:1900424, GO:1900150, GO:2000031, GO:0009617, GO:0009723, GO:0009620, GO:0009414, GO:0005829, GO:0005769	**4.14E-75**	**74.65%**
BT013052.1	SGN-U579408	2.162	proline rich protein	GO:0009536	1.67E-31	92.00%
TC217040	SGN-U593183	2.163	Acyltransferase	GO:0022900, GO:0009055	1.59E-38	85.60%
AK325938.1	SGN-U578266	2.186	avr9 cf-9 rapidly elicited protein 65	GO:0005739, GO:0003674	1.56E-17	57.00%
BM410860	SGN-U564294	2.190	hyphally-regulated cell wall protein	GO:0044413, GO:0044407, GO:0046658, GO:0009986	4.72E-05	42.86%
TA36518_4081	SGN-U581240	2.227	translationally controlled tumor protein	GO:0010252, GO:0009734, GO:0008283, GO:0042742, GO:0009819, GO:0009790, GO:0048527, GO:0007067, GO:0009860	1.74E-106	92.05%

mRNA/EST ID	UniGene ID	Fold Change	Gene Ontology Annotation	GO terms	Min. eValue	Mean Similarity
BG627276	SGN-U563117	2.241	Acyl-CoA binding domain containing protein 3	GO:0006629, GO:0006869, GO:0010029, GO:1900140, GO:0009737, GO:0009409, GO:0010288, GO:0010162, GO:0009505, GO:0005886, GO:0000062, GO:0032791, GO:0008289, GO:0070300, GO:0005515		
AK326023.1	SGN-U598751	2.277	nuclease harbi1-like	---NA---	0	73.05%
TA54887_4081	SGN-U575565	2.296	FMN binding	---NA---		
DB712015		2.297	BAP bon1-associated protein 2-like	GO:0019725, GO:0010286, GO:0009695, GO:0031348, GO:0010200, GO:0009409, GO:0009620, GO:0009408, GO:0009753, GO:0009612, GO:0009751, GO:0009266, GO:0009611	1.04E-48	66.85%
**AY348872.1**	**SGN-U600185**	**2.304**	**putative anthocyanin permease**	GO:0009901, GO:0009812, GO:0009555, GO:0016020, GO:0005886, GO:0005774, GO:0015297	**0**	**83.80%**

mRNA/EST ID	UniGene ID	Fold Change	Gene Ontology Annotation	GO terms	Min. eValue	Mean Similarity
AW928726	SGN-U600456	2.318	nc domain-containing protein	GO:0019725, GO:0009690, GO:0045087, GO:0060548, GO:0031348, GO:0009626, GO:0045793, GO:0010186, GO:0009270, GO:0009266, GO:0009697, GO:0009863, GO:0009627	4.84E-57	80.25%
AI777019	SGN-U597392	2.319	Sterile alpha motif SAM domain-containig protein	---NA---		
AK247916.1	SGN-U575586	2.320	domains rearranged methyltransferase	GO:0008757, GO:0032876, GO:0009825, GO:0010051, GO:0008610, GO:0008168	0	74.70%
TC205995	SGN-U565615	2.325	nitrate transporter	GO:0015112, GO:0009705, GO:0055085, GO:0015706	5.70E-41	89.45%
BI205317	SGN-U604418	2.344	delta-12 fatty acid desaturase	GO:0046471, GO:0005575, GO:0080167, GO:0006636, GO:0009507, GO:0052637	1.21E-72	88.90%
Z46674.1	SGN-U568616	2.362	hypothetical protein Pmar_PMAR016250 [Perkinsus marinus ATCC 50983]	---NA---	7.39E-04	62.00%
EG552887	SGN-U581921	2.390	exocyst complex component sec3a-like	GO:0008150, GO:0005829, GO:0000145, GO:0005886	3.33E-13	97.85%
AW094391	SGN-U597189	2.411	hypothetical protein SDM1_29t00007 [Solanum demissum]	GO:0007018, GO:0005524, GO:0003777, GO:0005874	1.56E-10	74.00%
GH622591	SGN-U586288	2.419	zinc finger	GO:0010200, GO:0005759, GO:0008270, GO:0009737	1.70E-48	60.55%

mRNA/EST ID	UniGene ID	Fold Change	Gene Ontology Annotation	GO terms	Min. eValue	Mean Similarity
TC198843	SGN-U583942	2.419	tir-nbs-lrr resistance protein	GO:0006915, GO:0006952, GO:0005524	8.59E-53	73.45%
AI772144	SGN-U593315	2.433	Transcription factor bHLH 125	GO:0005634		
TC211939	SGN-U566911	2.462	retrotransposon unclassified	---NA---	1.37E-14	60.95%
TC202803	SGN-U568616	2.462	protease do-like 7-like	GO:0005739, GO:0003674	3.09E-92	83.30%
TC211768	SGN-U573464	2.499	chlorophyll synthase	GO:0016020, GO:0009536	1.96E-07	56.88%
GO376376	SGN-U571241	2.542	nad -linked oxidoreductase-like protein	GO:0005886, GO:0016491, GO:0009536, GO:0016020	7.56E-107	89.60%
AW219006	SGN-U575716	2.567	calmodulin binding	GO:0017118, GO:0005516, GO:0005737, GO:0005886, GO:0006464, GO:0055085, GO:0005242, GO:0016415	1.38E-10	48.19%
AW039918	SGN-U583713	2.605	p27-like protein	GO:0008150, GO:0031225, GO:0005634	2.96E-103	69.35%
BI203929	SGN-U568616	2.635	merozoite surface antigen	---NA---	4.61E-11	53.05%
TC193612	SGN-U574954	2.635	cysteine proteinase	GO:0009740, GO:0009686, GO:0006508, GO:0009739, GO:0010114, GO:0005576, GO:0008234	0	70.05%
BG625890	SGN-U576640	2.656	atp synthase cf1 epsilon subunit	GO:0045261, GO:0042777, GO:0009851, GO:0008553, GO:0046933, GO:0005524, GO:0046961, GO:0009535,	1.10E-13	86.00%
AK327760.1	SGN-U564718	2.672	pi-plc x domain-containing protein at5g67130-like	---NA---	0	81.60%

mRNA/EST ID	UniGene ID	Fold Change	Gene Ontology Annotation	GO terms	Min. eValue	Mean Similarity
AK246885.1	SGN-U575511	2.685	at hook motif-containing protein	GO:0009734, GO:0009640, GO:0009723, GO:0008080	2.20E-29	64.85%
BE353618	SGN-U562988	2.846	golgi to er traffic protein 4 homolog	GO:0071816, GO:0005829	1.98E-65	78.50%
AW930418	SGN-U583556	2.896	receptor protein kinase	GO:0012505, GO:0004672, GO:0010204, GO:0010359, GO:0005515, GO:0005524, GO:0004674, GO:0009626, GO:0016301	5.44E-35	78.35%
BI203720	SGN-U575527	2.916	1-aminocyclopropane-1-carboxylate oxidase-like protein	GO:0046872, GO:0009693, GO:0006952, GO:0055114, GO:0009835, GO:0031418, GO:0009815	2.22E-45	77.00%
AK325661.1	SGN-U566912	3.247	retroelement pol polyprotein	GO:0004523, GO:0003677, GO:0006278, GO:0003723, GO:0009401, GO:0006310, GO:0051252	8.39E-51	47.60%
AY093595.1	SGN-U578601	3.272	osmotin-like protein	GO:0012505, GO:0005634, GO:0005737, GO:0051707, GO:0009651, GO:0009816, GO:0009817	0	94.55%
ES896576	SGN-U578580	3.861	calcium-dependent lipid-binding domain-containing protein	GO:0045892, GO:0009958, O:0009651, GO:0009414, GO:0005783, GO:0031965	3.47E-76	79.95%
DV105097	SGN-U580440	4.166	DNA binding protein 4	GO:0009536		
AK328540.1	SGN-U578955	4.532	flc-like 1 splice variant 4	GO:0003677, GO:0045449	1.01E-54	69.15%

Table S1. Differentially expressed genes annotation. The automatic BLAST annotation of sequences was performed by the BLAST2GO software suite and the fold change of transgenic plant compared to relative control cv Red Setter, expectation value (e value), sequence similarity and corresponding Gene Ontology terms were reported (---NA--- represents sequences not annotated, in bold were reported sequence validated by realtime RT-PCR -see Fig. S2-, in brackets we reported the name of the genes used in the paper)
